# Study on Collapse Resistance of RC Frame under the Corner Column Removal Scenario

**DOI:** 10.3390/ma14237157

**Published:** 2021-11-24

**Authors:** Jin Xu, Sheliang Wang, Kangning Liu, Xiaoyi Quan, Fangfei Dong

**Affiliations:** 1 School of Civil Engineering, Xi’an University of Architecture and Technology, Xi’an 710055, China; silent_xj@126.com (J.X.); sheliangw@163.com (S.W.); liukangning940310@163.com (K.L.); 18829247265@163.com (X.Q.); 2 School of Civil Engineering, Xijing University, Xi’an 710123, China

**Keywords:** 3D RC frame, progressive collapse, corner column removal, calculation for resistance, flexural action, compressive arch action

## Abstract

The progressive collapse of buildings induces a variety of catastrophic consequences, such as casualties and property loss over the past few decades. The corner column is more prone to abnormal load events compared to the inner column and outer column; thus, it is easier to trigger progressive collapse. By considering the effects of floor slabs and adjacent bays on progressive collapse behavior, the pseudo-static loading method was used to study the progressive collapse test of a 1/3 scaled, one story, 2 × 2-bay cast-in-place reinforced concrete frame substructure under the removal condition of a corner column. The test results show that the flexural deformation principally concentrates upon the components of a directly affected part (DAP), and compressive arch actions are observed in members of the indirectly affected part (IAP). Moreover, the slab adjacent to the removed column and periphery elements contributes great resistance to a progressive collapse.

## 1. Introduction

Progressive collapse refers to the partial failure of the main component, resulting in the collapse of the adjacent component and the collapse of the adjacent component, which further results in the collapse of the additional component [1,2]. The door to the investigation of the progressive collapse of structures was opened to the engineering community after the gas explosion at the corner of the Ronan Point Apartment Tower in London in 1968. In the past few decades, the progressive collapse of buildings induced a series of catastrophic consequences, such as casualties and property loss. Much research investigations have been conducted, and various design standards [3,4] have been proposed throughout the world in order to avert the progressive collapse of structures.

The probability of corner column failure is higher than that of interior or edge columns due to its particular position, which renders it vulnerable to abnormal load events. Consequently, progressive collapses triggered by corner column failure are more likely to occur. Furthermore, there is no external lateral restraint for the corner column with respect to the performance of RC structure with a corner column loss that would make a difference from ones with an interior or edge column loss. 

In order to figure out the response of progressive collapse induced by corner column failure in RC structures and to take efficient measures to hinder damage occurrence, a number of researchers have made much effort in this respect. Lim NS [5] conducted a group of contrast experimental tests of 3D RC substructure under corner column removal scenarios with a slab and without a slab. The results show that the presence of a slab could enhance the load-bearing capacity of structure. Qian K [6] proposed an analytical model validated by experimental results, and a comprehensive parametric study was carried out based on the proposed model in order to investigate the influence of several critical parameters. Yi [7,8] proposed a pseudo-static experimental method where the bottom column was replaced by a mechanical jack in order to simulate column failure, and a superstructure gravity load on the top of the failure column was simulated by a hydraulic actuator employing the force control method. 

Indeed, the corner column is one of the key load-bearing members in a building structure [9,10,11,12,13,14,15,16,17,18,19,20,21]. DoD 2010 [22] regulates that “for the frame structure, corner columns and exterior middle columns of long and short-side in the construction of each layer shall be removed for the collapse analysis one by one.” Even though a vast number of studies consisting of experimental [23,24,25] and numerical investigations [26] of progressive collapse have been conducted, the majority of studies with respect to progressive collapse subjected to scenarios of corner column loss pay more attention to plate-column structures. Few experiments investigated the response of progressive collapse under the scenarios of corner column loss for RC frame structure. 

In this paper, an experimental study on the behavior of 3D RC frame models containing slabs under a corner column removal scenario is presented with the help of a substructure technique. A progressive collapse is considered to occur if the remaining structures fail to redistribute vertical loads and the structural members reach the failure criteria after column loss. A more practical model was constructed and tested using pseudo-static experimental methods [27]. The test was fully instrumented, and indispensable measurements were in place for a comprehensive evaluation of responses throughout the entire process.

In the studies of progressive collapse of an RC frame structure, the influence generated by adjacent members is left out of consideration, and examining the bay-containing sectioned column takes up a great portion of studies. Progressive collapse is a global phenomenon, and the existence of slabs and adjacent bays might affect the behavior of progressive collapse. Thus, the study of progressive collapse should examine the response of integrated buildings that consist of columns, beams and slabs.

## 2. Experimental Program

### 2.1. Design and Fabrication of Specimen

In order to examine the performance of fully assembled 3D RC frame structures suffering from progressive collapse damage, the prototype structure on which the test specimen is based on is a 3-storey and 2 × 2 bay RC frame that was seismically designed in accordance with the Code for Design of Concrete Structures (GB 50010-2010) [28] and Code for Seismic Design of Buildings (GB 50011-2010) of China [29]. In the prototype, the RC two-bay slab is supported by longitudinal beams and transverse beams. The 400 mm square columns are spaced at a center-to-center distance of 5400 mm in the longitudinal direction and 3900 mm in the traverse direction. The story height is 3600, mm and the thickness of slab is 90 mm. The longitudinal beams have a 450 mm × 200 mm rectangular shape, while the transverse ones have a 350 mm × 200 mm rectangular shape. 

**Figure 1:**
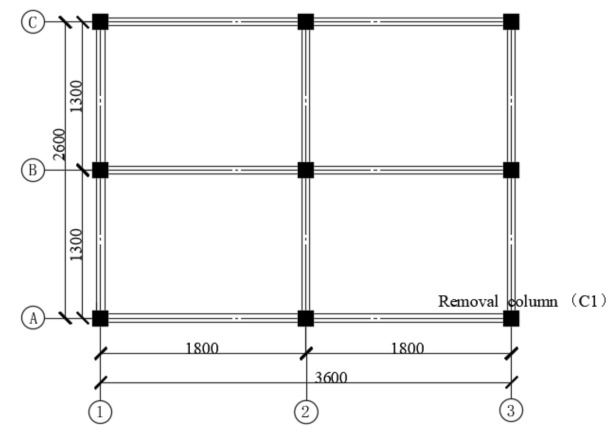
The typical two floor plan of the RC frame substructure.

Given the limited space in the laboratory as well as the limited loading capacity of actuator and experimental setup, a 1/3 scaled substructure extracted from the ground floor of the prototype was fabricated and tested. Figure 1 presents the typical floor plan of the 3D RC frame substructure; the equal distances between the longitudinal beams and the traverse beams were 1800 mm and 1300 mm, respectively. The thickness of slabs was 30 mm. The columns had reinforcing bars sticking out, and they penetrate the slab, which reached 90 mm above the top surface of the slabs and formed a column stub with a height of 100 mm, including a 10 mm concrete protective cover. The vertical continuity of the column was formed through a 100 mm high column stub fabricated for each column on the top of the slab. A 400 mm high bottom column stub underneath the slab was also fabricated at the removed column C1, which had a total height of 500 mm with respect to the failure column. The frame columns stirrups were encrypted throughout the height.

**Figure 2:**
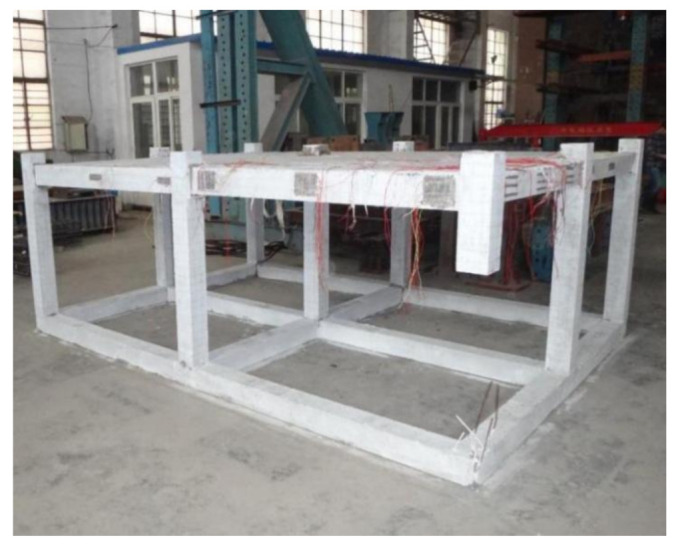
The aerial view of fabricated specimen.

Considering the contribution of the foundation to the stability of the entire frame structure, foundation beams were also constructed at the bottom of the specimen model. As in construction, the foundation beams were the same as the structural beams and all components were casted in one block. Meanwhile, a corner column (C1) was removed preferentially when the model was casted in place in the laboratory, but the corresponding frame joint was intact. In order to avoid initial deformations of the specimen as a result of the negation of the corner column, a timber pile was used as a support to simulate the failure column when pouring concrete. The aerial view of the fabricated specimen is presented in Figure 2.

### 2.2. Details of Steel Reinforcements and Concrete

**Figure 3:**
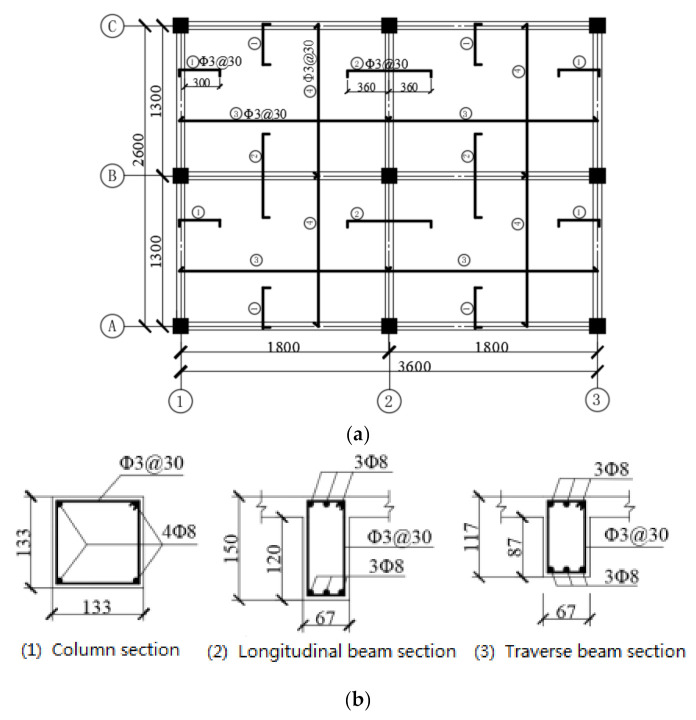
Details of slabs, beams and columns, (**a**) layout of slab reinforcing bars, (**b**) layout of beam and column reinforcing bars.

The component-reinforcing bars of the test specimen should be proportionately scaled down with reference to the prototype structure. By sticking to this principle, the specimen utilized the same reinforcement ratios as those of the prototype structure. Thus, steel HPB300 with the diameter of 8 mm was utilized for all load-bearing bars. For all stirrups in the specimen and distribution bars in slabs, however, no required reinforcement bar sizes were commercially available, and they were replaced by iron wire with a diameter of 3 mm. The grade of concrete used in the test is C25. Figure 3 demonstrates the slabs, beams and columns details, including reinforcement size, spacing and layouts.

In order to obtain mechanical properties, coupon tests were conducted for all selected materials. Two groups of concrete coupons (six concrete specimens) with 150 mm × 150 mm × 150 mm dimensions were reserved from test specimen and cured for 28 days; they were then tested for concrete cube compressive strength. For steel HPB300 and 3 mm iron wires, six sticks of coupon bars for each type were intercepted and tested in order to assess their mechanical performance index. Table 1 lists the mean values of the measured mechanical properties, where *d*, *f_y_*, *f_u_* and *E* represent diameter measurement, yield strength, ultimate strength and Young’s modulus, and *f_c_* corresponds to the compressive strength of the concrete cube, respectively.

### 2.3. Test Setup and Loading System

**Figure 4:**
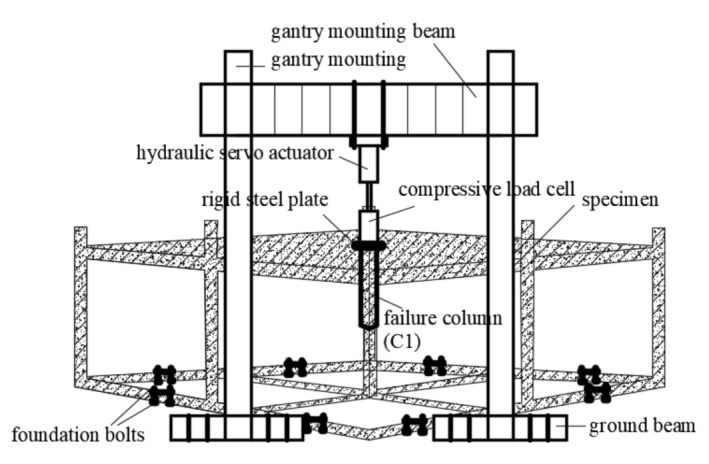
The test setup and RC specimen.

The test setup and specimen are schematically depicted in Figure 4. Assuming that the bottom joints of columns are performing as fully fixed ends, the foundation beams located at the bottom of the specimen were fixed on the ground by using strengthened anchor bolts.

A 500 kN hydraulic servo actuator, which was attached to the gantry mount by four long bolts, was loaded at the top of the failure column in order to simulate load applications by a superstructure. In order to obtain accurate resistance, a compressive load cell was placed below the actuator. A thick rigid plate was inserted between the load cell and the top area of the failure column in order to avoid crushing the concrete due to concentrated stress. According to Specificating of Texting Methods for Earthquake Resistant Building (JGJ101-96) [30], a mixed loading mechanism of force and displacement was selected for this experiment. In the initial loading stage, a constant vertical load was applied at the top of the failure column area until the specimen approached yield numbers. Following conversion to the displacement-controlled load method with a rate of at most 3 mm/min for specimen damage, progress was observed conveniently, and test data were obtained accurately. Referring to appendix B of DoD 2010 [23] where the frame beam loses its supporting column, the deflection at the supporting point should not exceed 10% of the beam span, which corresponded to 130 mm in this specimen. Therefore, the test was terminated when the applied displacement achieved the supposed maximum vertical displacement of 130 mm.

### 2.4. Instrumentation

**Figure 5:**
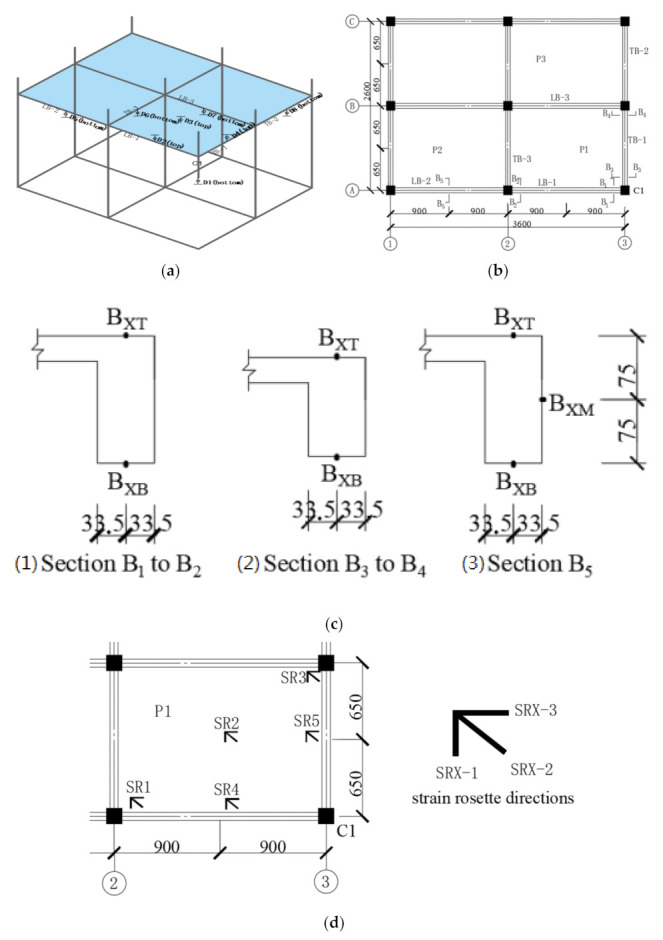
Layout of displacement and strain measuring points, (**a**) arrangement of displacement transducers, (**b**) strain gauge arrangements of beams (dimension unit: mm), (**c**) detailed locations of concrete strain gauges at the beam section level (dimension unit: mm), (**d**) strain rosettes arrangements of slab (dimension unit: mm).

Figure 5 illustrates instrumentation layouts, including linear variable displacement transducers, strain gauges and strain rosettes for the specimens. The resistance of the frame structure can be obtained from the compression load cell. A displacement transducer (D1) was placed at the bottom of the failure column in order to obtain its true displacement value. Three displacement transducers (D2–D4) were placed at the top, and four others (D5–D8) were placed at the bottom of slabs or beams in a vertical orientation. Seven strain gauges were distributed at three concrete sections (B_2_, B_4_ and B_5_) with two arrangement forms. Eight strain gauges, located at sections B_1_ to B_4_, were pasted to the rebar of the joint area where they might appear as plastic hinges prior to concrete crushing; prior to this procedure, the pasted gauges at every location were cleaned up and sand polished. Moreover, five strain rosettes (SR1–SR5) were arranged near the diagonal area of slab P1 in order to measure concrete slab strains in three directions.

## 3. Experimental Results and Discussion

### 3.1. Experimental Phenomena and Failure Mode

**Figure 6:**
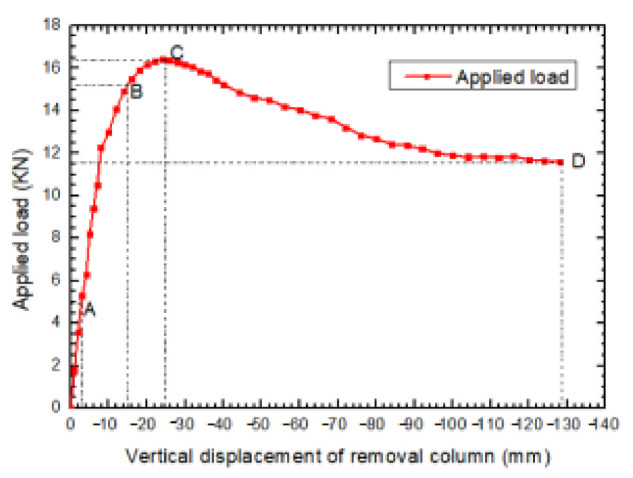
Static load-vertical displacement curve of the frame specimen with column removal.

**Figure 7:**
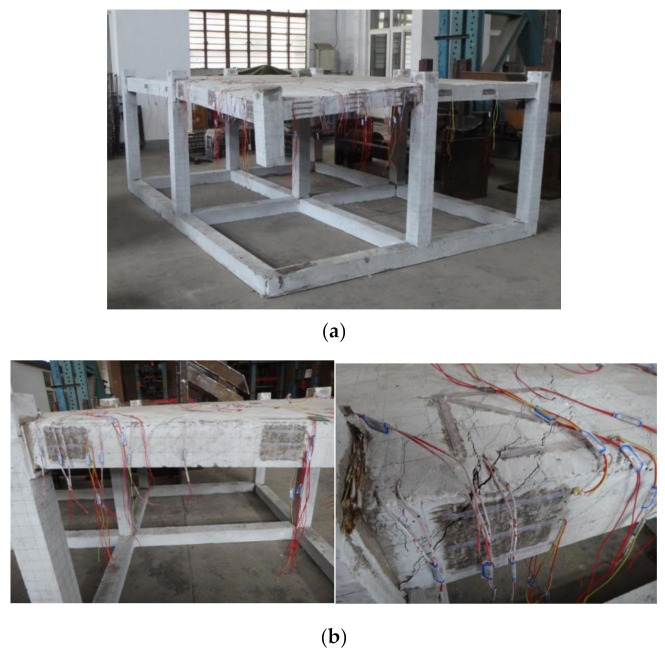

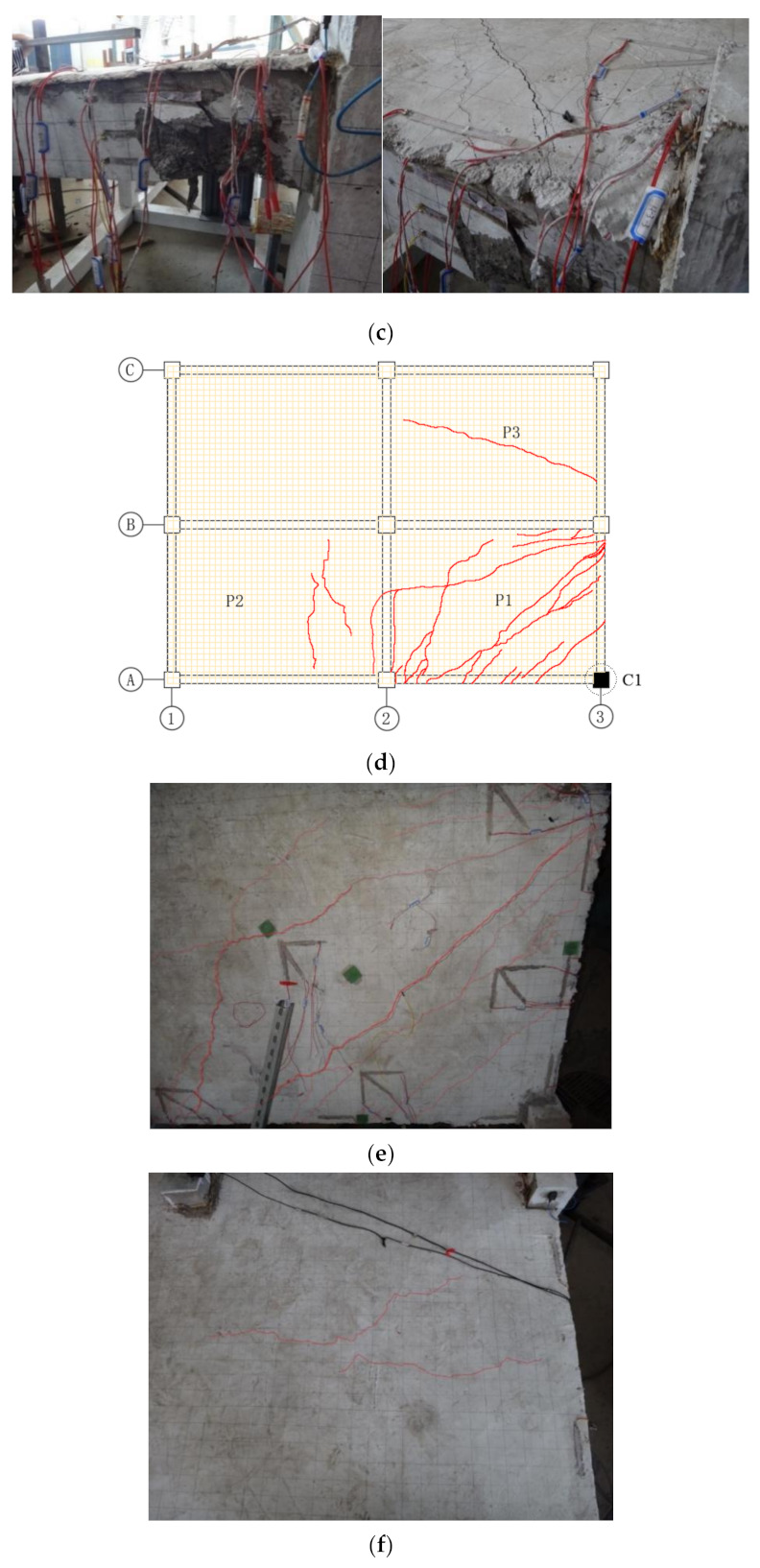

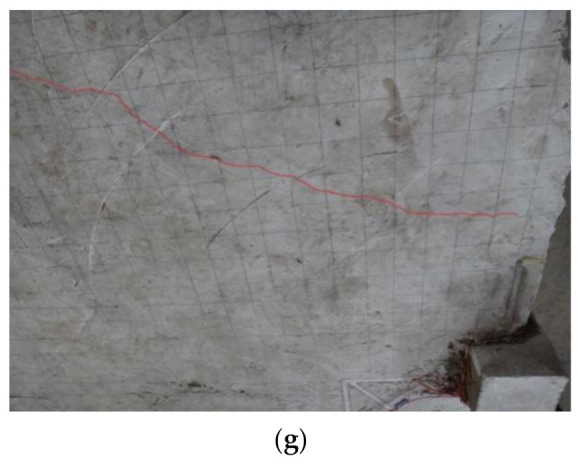
Overall views of the frame specimen’s deformed shapes at the final stage, (**a**) aerial view of frame specimen at the final stage, (**b**) deformed shape of beam LB-1, (**c**) deformed shape of beam TB-1, (**d**) crack pattern at top surface of slabs. (**e**) deformed shape of slab P1, (**f**) deformed shape of slab P2, (**g**) deformed shape of slab P3.

The relationship of applied load versus vertical displacement of the sectioned column in the specimen is displayed in Figure 6, in which the key stages during the test process are marked on the curves. Figure 7 presents overall views of deformed shapes of the specimen after the removal of the corner column. It is observed that the progressive collapse of the substructure occurs mainly in the area where the sectioned column is connected. Under monotonic vertical loads of the corner column, the specimen underwent large deformations and rotations prior to the final state. It can be concluded that the structure experienced three stages, compressing elastic stage, elastic-plastic stage and plastic stage, that increased the displacement of the failure column.

#### 3.1.1. Elastic Stage

Primitively, gradually increased force-controlled loading mechanisms were applied for the specimen, and the vertical displacement of the sectioned column kept rising. It is obvious that the state of the specimen was elastic without evident deformation. The ratio of structural resistance and displacement of the sectioned column, as well as structural stiffness, was kept approximately constant. However, at the moment when vertical displacement was about 2.6 mm (point “A”) and resistance was about 5 kN, flexural cracks appeared firstly at the further ends of the top area of the transverse beam (TB-1) that was adjacent to the sectioned column, which symbolized the end of the elastic stage. 

#### 3.1.2. Elastic-Plastic Stage

In this stage, the load mechanism was converted from the force-controlled method to the displacement-controlled method. Plastic deformations were consequently initiated, and the specimen’s resistance continuously increased; however, structural stiffness did not significantly decrease with respect to increasingly applied displacement. Then, more flexural cracks developed and were widened step by step at the further ends (B_2_ and B_4_ are beam sections) of the top area of the frame beams (LB-1 and TB-1). With the increase in applied displacement, LB-1 and TB-1 performed a twist shear deformation towards the slab’s center point, which resulted in slanting cracks appearing at the beam ends. Excessive concrete crushing and spalling occurred at the frame beam’s farther ends, and the steel bars distributed at this joint were severely exposed. At the same time, multiple slanting cracks running at 45° emerged and expanded and were rapidly accompanied with yield line cracks at slab P1’s top surface, and these cracks penetrated through the longitudinal and traverse beams. In addition, two and one slender cracks were, respectively, discovered at the center regions of the top surfaces of slab P2 and P3. When vertical displacement was about 16 mm (point “B”), where the resistance was about 15 kN, the plastic hinges at section B_2_ of LB-1 and B_4_ of TB-1 basically formed. Thence, the structural stiffness exhibited significant degradation; consequently, resistance increased quite slowly under increasing vertical displacement of the sectioned column. A maximum peak load of 16.4 kN was reached at a vertical displacement of about 25 mm (point “C”), and the plastic hinges located not only on the longitudinal beam but also the transverse beam ends had formed completely; moreover, the yield lines were adequately formed at the slab’s top surface, which denotes that the beams and slabs attained their flexural abilities, and this stage came to an end. The crack pattern represented the flexural action that played a leading role prior to peak applied displacement. 

#### 3.1.3. Plastic Damage Stage

After the maximum peak load, the deformation of end regions in frame beams and the vicinity of the diagonal in slab P1 became larger and larger and was accompanied by partially crushed concrete and continually spalled off; this resulted in frame resistance that presented critical degradation, although the vertical displacement of the sectioned column increased and gradually converged to a constant value of 12 kN (73% of the peak load) approximately when it approached the end of the test with a displacement about 130 mm (point “D”). An ever-decreasing load denotes the absence of a mutual stretch effect of components at the final stage. However, it is obvious that the specimen no longer had enough capacity to resist progressive collapse when vertical displacement reached 10% of the frame beam’s span, which is defined as failure displacement when referring to DoD 2010 [23]. It is a considerable deformation value for the progressive collapse of RC frame structure, defined in DoD 2010 [23].

### 3.2. Vertical Displacements of Frame Components

**Figure 8:**
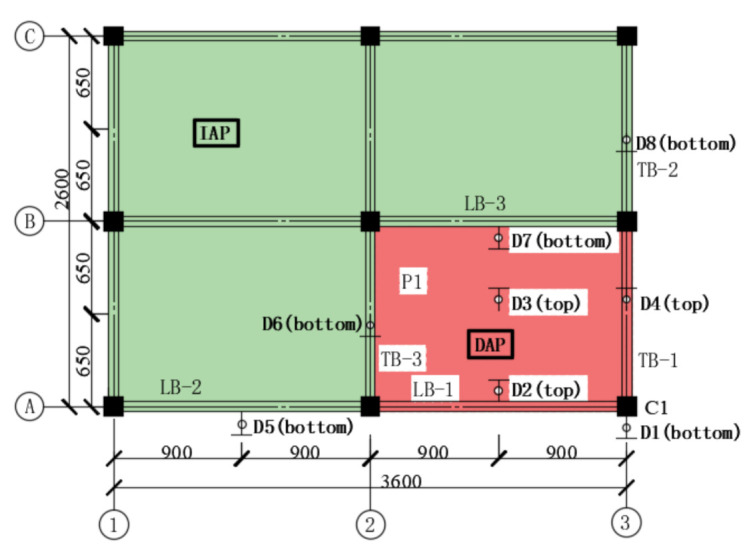
Definition of DAP and IAP; red color is indicative of DAP, and green represents IAP.

According to Specificating of Texting Methods for Earthquake Resistant Building (JGJ101-96) [30], a frame can be divided into a directly affected part (DAP) and an indirectly affected part (IAP) when a column is lost in the frame. DAP represents the part of the building that is directly affected by column loss; similarly, IAP represents the part of building that is indirectly affected by the force developing in the directly affected part. The specimen in this test could be divided into DAP and IAP, as pictured in Figure 8.

**Figure 9:**
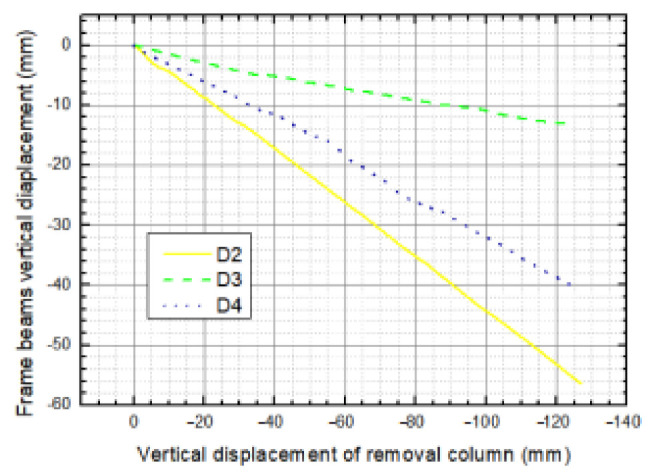
The vertical displacement of the DAP components.

In order to explore the deformation of the frame beams of DAP with respect to the response of progressive collapse after corner column loss, vertical displacements in the middle span of beam LB-1 (D2), TB-1 (D4) and slab P1 (D3) were measured by using vertically arranged displacement transducers. The vertical displacements of the LB-1, TB-1 and P1 along with applied vertical displacement are demonstrated in Figure 9**,** where negative values represent downward movement; in contrast, positive values are indicative of upward movement. It is observed that the components exhibited quintessential elastic deformations under small displacements. With increasing displacements of the sectioned column, movements developed in DAP members were not strictly linear, which implies that the plastic hinges were basically formed in the joint region. From Figure 9, it can be observed that the increased rate of vertical displacement at D2 obviously exceeded the one at D4; the value of D2 reached 56 mm (0.44 D1), while D4 approached 40 mm (0.32 D1) at the end of the experiment. This phenomenon is attributed to the stiffness of TB-1, which is superior to LB-1 due to the former’s shorter span. Furthermore, the vertical displacement of P1 is invariably between LB-1 and TB-1.

**Figure 10:**
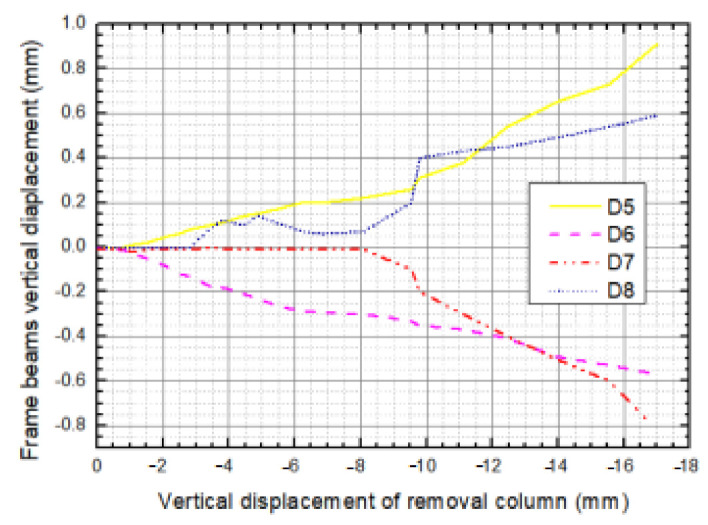
The vertical displacement of the IAP components.

Load distribution took place in the structure building following a certain structural component failure and force-carrying capacity disparity. In order to examine the impact of IAP components, four frame beams (LB-2, LB-3, TB-2 and TB-3) indirectly connected to the sectioned column were measured at the middle span for their vertical displacements. According to Section 3, we demonstrated that the resistance of frame substructure begins to decrease due to a host of plastic hinges; consequently, the deformation of IAP components varies in minor amplitudes. Figure 10 demonstrates the curves of vertical deformation measurements of D5-D8 versus the vertical displacement of the sectioned column within the premier loading displacement of 17 mm. It is observed that beam LB-2 and TB-2 moved upward while LB-3 and TB-3 moved downward; in particular, the beams indirectly connected to the sectioned column through the frame beam moved upward, but those indirectly connected to the sectioned column through the slab moved towards the contrary direction. The reason behind this discrepancy is the difference in resistance mechanisms that exist in the beams and slabs. The absolute values of vertical displacement of all four beams generally increased with increasing loading displacement. In particular, LB-3 stayed at its original position without any movement until loading displacement approached 8 mm. Moreover, TB-3 exhibited considerably deformed tendencies when displacement started to increase from the loading displacement of 2.6 mm; however, when the displacement reached 1.2 mm, a transient retraction occurred and increased quickly. In addition, a significant retraction appeared when loading displacement was around 5 mm after recovering, and tencdency increased until a loading displacement of around 8 mm was attained. Combined with the test process that investigated what triggers this abnormal phenomenon, a possible reason behind this is that the concrete split and fell off, and a portion of energy that had been transferred into the structure was released; consequently, the strain energy of the structure decreased in proportion.

### 3.3. Strain Evolution, Distribution and Internal Force from Strain Measurements

From the test photos and above analysis, we can observe that the damage of materials and components was primarily gathered at the DAP; hence, the strain and stress evolutions will pay more attention on DAP elements.

**Figure 11:**
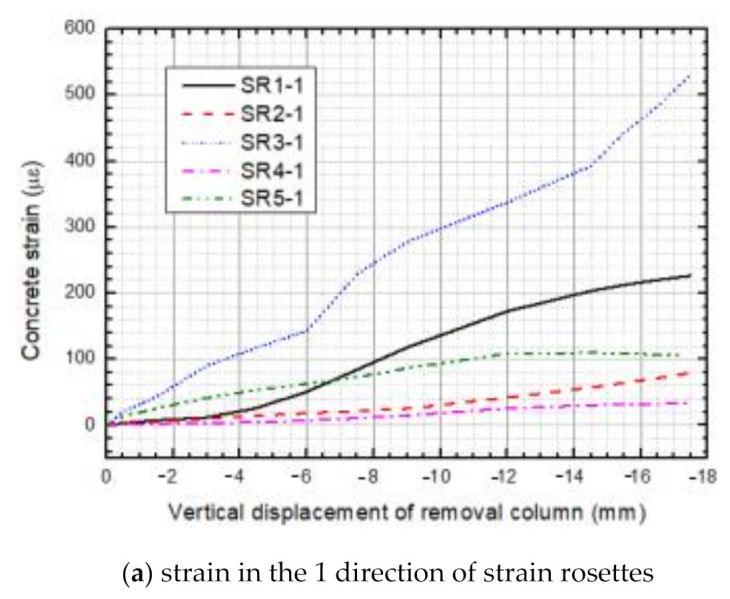

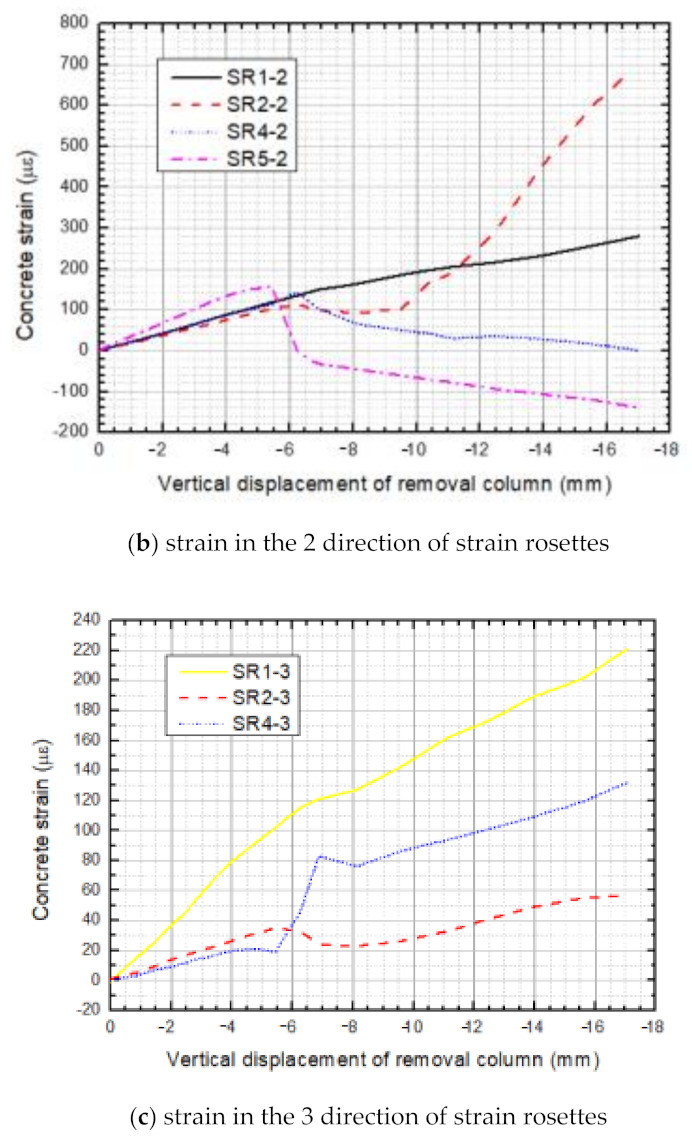
Concrete strain at top surface of P1. (**a**) strain in the 1 direction of strain rosettes; (**b**) strain in the 2 direction of strain rosettes; (**c**) strain in the 3 direction of strain rosettes.

The floor slabs existing in 3D RC frame structure can enhance the strength of the beam-column joints and improve force-bearing capacities of the structure significantly. Under the applied load, however, the floor slab that was connected to the failure column could be, in turn, subjected to load action. Two-way slabs, conveying superstructure loads to beams and columns in two perpendicular directions, were employed in this test specimen; thus, strain rosettes at three directions were utilized to record strain development in slab P1. Stress distribution and development in P1 could be inferred from the curves of strain versus vertical displacement of the sectioned column plotted in Figure 11. Since the cracks crossed through certain strain rosettes, the stress state cannot be accurately reflected by later measurements; thus, the first 17 mm strain-vertical displacement curves are the only ones presented. Moreover, three strain evolutions were absent because of errors in the gauges, and their reliable measurements could not be obtained.

It is easily observed that the three directions of concrete strains SR1, SR2 and SR4 have positive values, which means that the concrete at the top surface of P1 is in tension. Moreover, the tension force directed to the sectioned column coped with the test phenomenon in Section 3.1. In the one and three directions, strain values of five gauging points were positive, which implied that concrete of the five positions on slab P1 was in a state of tension in these directions. Among them, SR3-1 obtained the maximum strain and surpassed the others in the one direction during the loading process, particularly where the maximum tension force bearing by the slab was assessed. Similarly, SR1-3 obtained maximum strain in the three direction throughout the loading process, particularly where the maximum tension force bearing by the slab was assessed. What is noteworthy is that, in the 2 direction, SR2-2, SR4-2 and SR5-2 demonstrated positive strain decrease despite SR2-2 obtaining an increased value, but the other strains kept decreasing; even the sign of strains in SR5-2 was reversed at a vertical displacement of about 6 mm. One reason behind the above variation is that, along with the increasing vertical displacement of the sectioned column, when either longitudinal or traverse beams bend in their frame plane, the distance between two ends of one beam becomes shorter; as a result, the longitudinal beam torsion is directed towards the traverse axis, while the traverse beam is directed towards the longitudinal axis, and the longer the distance of a section from the sectioned column, the greater the torsion displacement corresponding to concrete compression forces at the section. Therefore, a reduction in SR4-2 and SR5-2 strains continuously developes and exceeds others.

**Figure 12:**
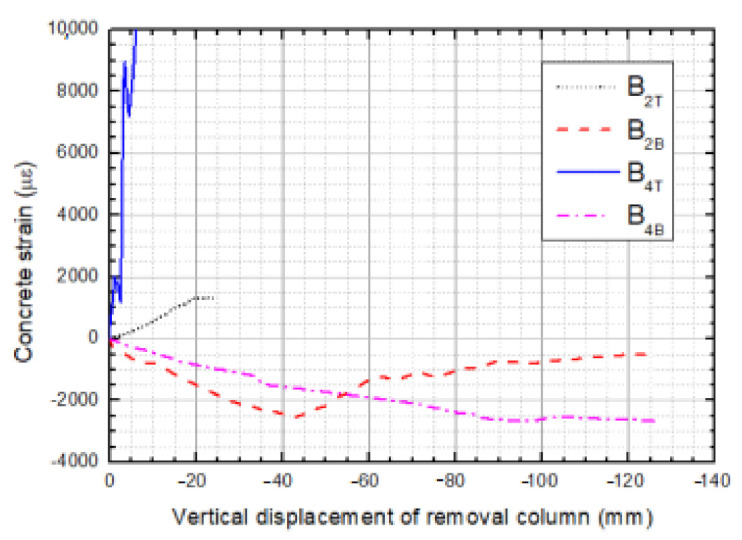
Concrete strain at B2 and B4.

Concrete strains in the axial direction at the vital beam sections are rendered in Figure 12. The selected sections are located at the farther ends of the beam relative to the sectioned column and can reflect the maximum bearing-force status of concrete in the specimen. Taken together, with increasing applied displacement, the concrete at the top surface is in tension, while those at bottom surface experience compression. Concrete at the bottom of section B_4_ of TB-1 foremost split due to unbearable tension forces when the applied displacement was at 2.6 mm; thereafter, the concrete tensile strain continues to soar but had no consequences. The occurrence of the first crack momentously symbolizes the end of the elastic stage of the structure. Concrete at the top surface of section B_2_ of LB-1 also split when the applied displacement reached up to about 4.5 mm, and a crack appeared through the strain gauge, later resulting in unreliable strain. Concrete that is located at the bottom surface of these sections was experiencing compression throughout the process, and crush concrete even spalled off when displacement exceeded the ultimate strain.

Table 2 lists the concrete strain evolution at the middle-span sections of B_5_ of LB-2. It is explicit that concrete at the top surface is in tension, but those at the middle and bottom surfaces are in compression. This bearing-force pattern existing in LB-2 appears as an arch, which demonstrates compressive arch action. A deduction could be made that compressive arch action developed as well in the beam TB-2. In fact, the vertical displacement of TB-2 assessed by D8 verifies that compressive arch action did exist even though it experiences retraction for a while.

**Figure 13:**
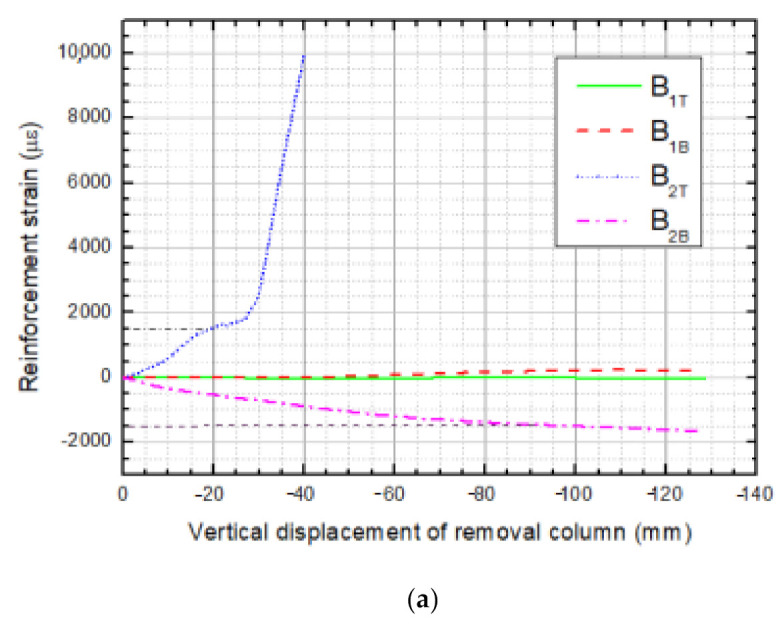

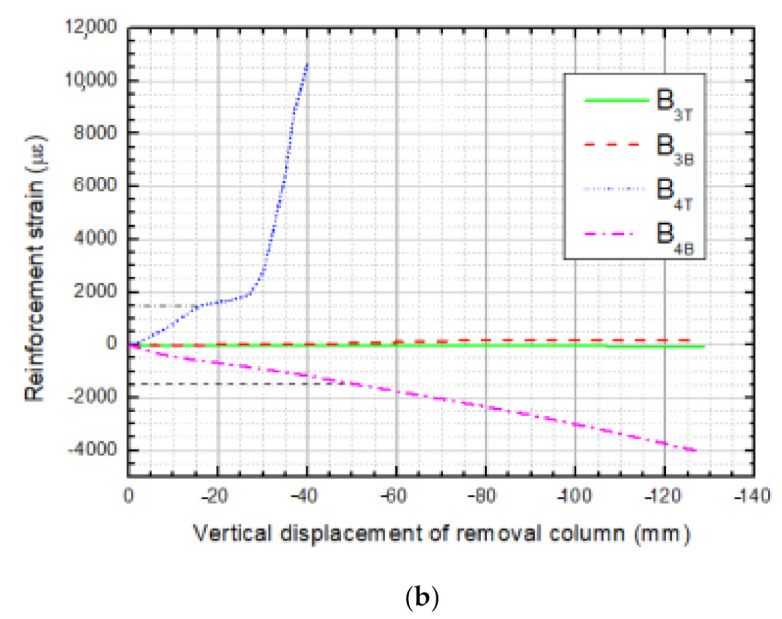
Reinforcement strain of beams in DAP, (**a**) reinforcement strain of beam LB-1, (**b**) reinforcement strain of beam TB-1.

The absence of bending moments at DAP beam ends could be inferred from the development of reinforcement strains of LB-1 and TB-1 sections illustrated in Figure 13a,b, respectively. As load gradually proceeded from zero to the peak displacement corresponding to the peak load, the top reinforcement at section B_4_ of TB-1 reached the tensile yield strain (1490 με) at first when the applied load displacement was about 15 mm, which lagged behind the loading displacement of crushed concrete; thereafter, steel bars at section B_2_ at the top of LB-1 reached the tensile yield strain almost simultaneously. Subsequently, plastic rotation came up in these sections, and plastic hinges formed basically along with increasingly applied displacement. A growing number of plastic hinges were formed by degrees in other sections until the structure could not sustain greater force. it is observed that, in the total loading process, top reinforcements experience ever-increasing tension, while bottom ones experience ever-increasing compression; from this, it is inferred that bending moments exist in the beam’s overall process, and there is no axial tension force in the beam along the span direction. In addition, compressive reinforcement yields occurred behind the peak load. The reinforcement strains at section B_1_ reach almost zero values, which reveals that the beams and slabs behaved as cantilevers under a corner column removal scenario. Furthermore, only flexural action operates, but no catenary action exists in DAP beams in this case.

## 4. Calculation for Ultimate Resistance to Progressive Collapse

**Figure 14:**
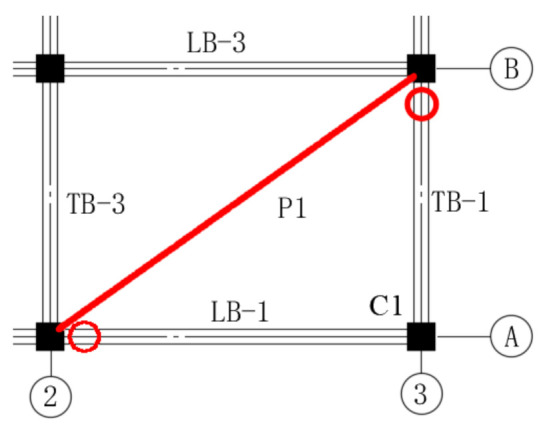
Positions of plastic hinges in beams and yield line in slab.

The experimental results reveal that plastic hinges (line) mainly appeared in the positions illustrated in Figure 14 when the structure was subjected to progressive collapse due to the failure of the corner column. That is to say that the load applied on the failure column top is mainly resisted by the resisting bending moment of these sections; thus, the structure’s resistance to progressive collapse could be estimated by calculating the sum of each ultimate bending moment of these sections.

### 4.1. Calculation for Resistance Supplied by DAP Beams

Beams LB-1 and TB-1 are foremost severally damaged, and plastic hinges had formed in the farther ends of the sectioned column, which means that the ultimate flexural strength had achieved in these sections. Thus, the applied vertical load prompted the attainment of full plastic yield moments at section B_2_ of LB-1 (F_l_) and section B_4_ of TB-1 (F_t_), respectively, and can be calculated as follows:

(1) $$F_{1}\times L_{1}=M_{1}$$

(2) $$F_{t}\times L_{t}=M_{t}$$

where M_l_ denotes the ultimate yield moment of LB-1, and M_t_ represents the ultimate yield moment of TB-1. L_l_ and L_t_ are the longitudinal and traverse beam’s length, excluding column width, respectively. The ultimate yield loads F_l_ and F_t_ are calculated by substituting the relevant parameters, which are 3.65 kN and 3.88 kN respectively.

### 4.2. Calculation for Resistance Supplied by DAP Slab

The slab of 3D RC frame substructure has also played a critical role in resisting progressive collapse. For slab P1, the yield line theory for the ultimate flexural strength of RC slabs proposed by Hognestad E^18^ was employed. The ultimate bending moment resistance per unit width in the slab yield line (m_u_) can be calculated as follows:

(3) $$m_{u}=A_{s}f_{y}(d_{s}-\frac{A_{S}f_{y}}{1.6\;f_{c}})$$

where A_s_ is the area of tension reinforcement per unit width of the slab, f_y_ and f_c_ are the tensile yield strengths of reinforcement and compressive strengths of concrete, respectively, and d_s_ stands for the effective thickness of slab.

Assuming that F_s_ is the ultimate load applied at the failure column and δ is the corresponding vertical displacement and according to the principle that states that external force work equals internal work, an equation can be listed as follows:

(4) $$F_{s}\times \delta =m_{u}\times L\times \frac{\delta }{L/2}$$

where L is the length of negative moment yield line equated to the diagonal of the slab, and $\delta /\left(L/2\right)$ is the rotation of the slab’s hogging moment. According to the layout of steel bars in the slab shown in Figure 3a, the calculated ultimate yield load (F_s_) by substituting m_u_ as 2.56 kNm is 5.12 kN.

### 4.3. Calculation for Total Resistance Supplied by DAP Components

Pc is defined as the calculated structure resistance provided by DAP members by summing Fl, Ft and Fs, which equals 12.65 kN (79% of the peak load); meanwhile, we note that the calculated resistance of a slab accounts for 40% of the total calculated structure resistance, which confirms flexural action playing a core role under a progressive collapse scenario caused by corner column loss. One reason behind the different values is that IAP members also supplied partial resistance by accumulating permanently deformed energy, for example, LB-2, LB-3, TB-2, TB-3, P2 and P3; another reason is that there are several regions that resist the applied load and not only one section in a component. Last but not least, there exists mutual effects that could enhance the structure building’s resistance rather than a simple superposition of resistance offered by each member.

## 5. Analysis of Progressive Collapse Resistance Mechanisms

No matter what damaged pattern occurs, progressive collapse incidents all begin with large deformation failures of damaged positions. Consequently, resistance mechanisms that exist in the components are crucial for opposing progressive collapse. By lacking external lateral restraints, no catenary actions were observed in 3D RC frame substructure specimen under a corner column loss scenario; instead, flexural action and compressive arch action were observed.

### 5.1. Flexural Mechanism

The flexural mechanism has been verified as a notable factor in resisting external load. Section 3 and Section 4 have suggested that structural beams and slabs of DAP behaved as cantilevers and resisted the applied load at the sectioned column’s top by means of flexural moment resistance. Once the resistance moment at all sections reached its ultimate resistance moment, however, the structure’s resistance did not experience extra increments during the large deformation stage in the corner column-removed test.

### 5.2. Compressive Arch Mechanism

Compressive arch mechanisms usually appear in the structural beams of DAP during the small deformation stage under an interior column loss scenario. However, for a corner column loss case, compressive arch mechanisms are presented in members of IAP but not DAP. With the addition of slabs in this experiment, slight compressive arch action emerges in the slabs of IAP parallel to the beams of IAP due to the end moments distributed by the joint.

The corner column is inclined towards the interior of the structure in this specimen. In fact, due to the restraints of the upper column in a real RC frame structure building when it is subjected to corner column loss damage at the large deformation stage, the tilt tendency of the corner column stub will be constrained, and more sections in the joint region above the sectioned column will attain their ultimate resistance bending moments. In this case, an improvement could be obtained for both load-carrying and deformed capacities of structure. This action existing in structures is named the Vierendeel mechanism. Unfortunately, only a one-story test specimen was constructed; consequently, the Vierendeel mechanism could occur, and this resulted in an underestimation of the resistance of the test specimen. In future experimental examinations, the Vierendeel mechanism in a 3D RC frame structure building needs to be further investigated.

In summary, flexural mechanisms, compressive arch mechanisms and Vierendeel mechanisms all contribute to the resistance of a 3D RC frame structure building. In small deformation stages, flexural mechanisms and compressive arch mechanisms mainly have the effect of resisting progressive collapse, while when it comes to large deformation stages, the Vierendeel mechanism resists progressive collapse.

## 6. Conclusions

In this paper, an experimental test was conducted and aimed at investigating the progressive collapse performance of a 3D RC frame structure by using pseudo-static experimental methods with the help of substructure techniques. Firstly, deformation and the failure mode of the substructure were studied in detail. Then, the calculation for the resistance of the specimen was carried out, and three reasons behind the deviation value were analyzed. Finally, resistance mechanisms of 3D RC frame structure in different stages of progressive collapse are discussed. The following conclusions could be drawn:

1. Under monotonic vertical loading on the corner column, the specimen underwent three stages, including compressing elastic stage, elastic-plastic stage and plastic damage stage.
2. Crack patterns developed in the frame specimen. Flexural cracks appeared in slabs P1, P2 and P3. In particular, both flexural cracks and twist cracks emerged in the beams of DAP. Plastic hinges firstly appeared in the farther end of TB-1 because of its greater stiffness and finally formed in the farther ends of LB-1 and TB-1. A plastic hinge line was completely acquired in the diagonal of P1.
3. The calculated resistance was approximately 79% of the experimental peak load, which is possibly due to the three following reasons: the contributions of deformed energy of IAP members, the increased sections yield of beams in DAP and the mutual effect between all components. The calculated resistance of the slab contributes greatly to the total structure’s resistance.
4. Flexural mechanisms of members in DAP and compressive arch mechanisms of components in IAP are mainly resistance mechanisms in the progressive collapse of structures under a corner column loss scenario. Moreover, the Vierendeel mechanism at large deformation stages in 3D RC frame structure needs to be further investigated in the future.

## Figures and Tables

**Table 1:** Properties of steel bars and concrete.

Material		*d*, mm	*f_y_*, MPa	*f_u_*, MPa	*E*, MPa
Steel bars	HPB300	8	310	372	2.08 × 10^5^
	Iron wire	3	395	450	2.01 × 10^5^
		*f_c_*_,_ MPa		*E*, MPa	
Concrete	C25	26		2.81 × 10^4^	

**Table 2:** Concrete Strain at middle-span section of LB-2.

Loading Displacement, mm	0	11.12	22.16	31.2	35.21	41.7	51.5	83.4	127
B_5T_	6	54	88	110	111	111	112	114	115
B_5M_	−3	−33	−57	−68	−74	−76	−79	−87	−102
B_5B_	−12	−133	−220	−269	−283	−288	−296	−319	−334
